# Effects of Arts-Based Pedagogy on Competence Development in Nursing: A Critical Systematic Review

**DOI:** 10.3390/nursrep14020083

**Published:** 2024-04-27

**Authors:** Berit Sandberg

**Affiliations:** HTW Business School, University of Applied Sciences Berlin, 10318 Berlin, Germany; berit.sandberg@htw-berlin.de

**Keywords:** arts-based learning, arts-based pedagogy, arts-based teaching, competence development, evidence-based practice, nursing education

## Abstract

The integration of arts-based methods into nursing education is a topic of growing interest in nursing practice. While there is an emerging body of research on this subject, evidence on competence development remains vague, largely due to methodological weaknesses. The purpose of this review is to evaluate the effectiveness of arts-based pedagogy in nursing, specifically in terms of students’ changes in knowledge, skills, and attitudes. It explores which arts-based approaches to nursing education qualify as evidence-based practice in terms of nursing competence. A systematic critical review of research on arts-based pedagogy in nursing was conducted, identifying 43 relevant studies. These studies were assessed for methodological quality based on the CEC Standards for evidence-based practice, and 13 high-quality comparative studies representing a variety of arts-based approaches were selected. Creative drama was identified as the only evidence-based practice in the field, positively affecting empathy. The findings highlight a research gap in nursing education and emphasize the need for measurement and appraisal tools suitable for the peculiarities of arts-based pedagogy.

## 1. Introduction

Nursing has been described as both an art and a science since Florence Nightingale’s influential work, ‘The Art of Nursing’, was published in 1859 [1]. The artistic aspect of nursing has been a topic of discussion in the field of education for many years [2,3,4,5,6]. Scholars have complemented this concept by integrating liberal arts into nursing education [7,8,9,10]. This concept has been supported by professional associations [11] and global policy recommendations [12].

The inclusion of arts and humanities in the training of healthcare professionals aims to enhance learners’ competencies in clinical and personal skills [12]. In nursing education, the arts and humanities help learners comprehend and appreciate human experiences. Some argue that knowledge of aesthetics can improve nurses’ imaginative abilities and provide a more holistic understanding of themselves, human nature, and the caregiving process [13,14,15,16].

The integration of arts-based methods into nursing education has gained interest due to the growing recognition of the importance of a holistic approach to nursing [17,18]. This development aligns with the demand for competency-based education within curricula [12,19] and learner-centered approaches in the classroom, such as experiential learning [20].

In the sense of teaching *through the arts*, arts-based methods are a subfield of aesthetic teaching or aesthetic learning alongside teaching *about* and *in the arts* [15,21,22]. However, there is no appropriate term for using the arts as a didactic element. Common terms used in the literature include “arts-based learning” [23], “arts-based teaching” [24], “arts-based education” [25], and “arts-based pedagogy” [26,27]. These hyphenated terms highlight the interdisciplinary nature of arts-based teaching and learning, while also distinguishing it from artistic education and art pedagogy [28].

Arts-based pedagogy is a creative strategy that uses an art form to facilitate learning about another subject matter ([23], p. 53). This approach goes beyond decorative or entertaining elements in the classroom, such as background music (e.g., [29]). Learners engage with artistic works, perform them, or create their own. In this process, engagement with at least one art form, such as visual and performing arts, music, or literature, can aid in the acquisition of knowledge or skills in non-art subject areas [21,23,26]. Arts-based pedagogy facilitates experiential learning by considering sensory experience and aesthetic reflection as independent sources of knowledge and cognition [15,21].

Integrating arts and creative approaches into nursing education encourages students to explore beyond the traditional scientific and technical aspects of nursing and to engage with the emotional, social, and cultural aspects of the nursing profession. Arts-based approaches can increase learners’ involvement, motivation, and attention by drawing from their experiences and creating an emotional connection to difficult topics [26,27,30,31]. This pedagogy complements training that primarily focuses on cognitive and psychomotor learning goals by addressing the affective level of learning [23,26,32].

Nursing practice requires a complex set of competencies, including clinical skills, interpersonal abilities, and humanistic practice [33]. Arts-based pedagogy has been used to address many of these competencies. Researchers suggest that arts-based nursing education can assist future nurses in developing a professional identity. The arts are believed to enhance critical thinking, diagnostic skills, and communication abilities. Enhancing empathy toward clients contributes to improved patient care quality and patient-centered nursing [30,34,35,36,37,38,39]. Additionally, arts-based approaches have been recognized to strengthen nursing students’ resilience and help them cope with the high stress levels associated with the nursing profession [40,41].

Conceptual papers and empirical research generally present a positive view of arts-based nursing education and its effects, as reflected in relevant reviews. They either cover the entire field [27,32,42,43,44] or explore the integration of different genres within nursing education, such as the visual arts [31,45,46], drama [47,48], poetry [49,50,51], storytelling [52], and film (cinenurducation) [53,54].

However, many studies exploring the impact of art-based pedagogy in nursing education lack methodological quality and rigor. Most studies are qualitative and do not define what makes an intervention successful. It is suggested that qualitative studies may overestimate learning effects, while the actual development of competence may be lower than what a positive evaluation of arts-based learning experiences suggests [55]. In the case of quantitative research on arts-based pedagogy in nursing education, uncontrolled studies with limited internal validity are prevalent [56]. Outcome measures in many cases are not robust because they rely on participants’ self-assessment [43,57].

A rigorous evaluation of arts-based nursing education is necessary to determine its impact on learners’ knowledge acquisition, skill development, and attitudinal changes. Previous reviews have not systematically addressed this issue. Quantitative intervention studies are crucial in educational impact research because they allow for statistical verification of the causality between intervention and effect. They are an essential element of evidence-based practice (EBP), where the effectiveness of an intervention is the determining factor [58]. A practice is considered evidence-based if it is “supported by a sufficient number of research studies that (a) are of high methodological quality, (b) use appropriate research designs that allow for assessment of effectiveness, and (c) demonstrate meaningful effect sizes” ([59], p. 495).

This systematic review aims to determine if arts-based nursing education meets the criteria for evidence-based practice (EBP). It examines the extent to which rigorous research has been conducted on arts-based pedagogy in nursing, with attention to research design, methodological quality, and effect size [60,61,62]. As a critical review, this study explores the quality and credibility of quantitative research on arts-based nursing education. It aims to uncover potential methodological flaws or bias, make recommendations for future research, and inform practice in the field [63,64]. The paper takes a systematic approach to explore the impact of arts-based interventions on competence development as reflected in quantitative research. What are the reported effects on knowledge, skills, and attitudes resulting from art-based interventions? Is there scientifically robust research demonstrating their effectiveness [64]? The purpose of this review is to assess the effectiveness of art-based pedagogies in nursing and to support the concept of evidence-based nursing education [65].

## 2. Materials and Methods

This review follows the methodological approach for conducting systematic reviews as outlined by Kitchenham and Charters [66]. The approach includes the following stages: study selection, identification of research, quality assessment, data extraction, and data synthesis. The protocol for this systematic review was registered on INPLASY (INPLASY202440071).

### 2.1. Inclusion Criteria

#### 2.1.1. Phenomena of Interest

As research on nursing education is the context of this study, the review encompasses all forms of training and development for nursing professionals, including secondary education in nursing degree programs and professional development. Secondary education in nursing degree programs as well as professional development are considered. The review also includes studies in which the participants were not exclusively nursing students or professionals.

This review focuses on arts-based pedagogy in nursing education. It includes studies in which learners receive works of visual art (painting, sculpture, graphics, photography, performance and media art, etc.), performing arts (theater, dance), music, film, or poetry. It also includes studies in which learners themselves create artifacts or actively engage in creative expressions such as theater, dance, narrative storytelling, etc. [67]. The review excludes methods that are not considered arts-based, such as photovoice, concept mapping, reflective writing, and standardized patient simulation using drama students. It considers interventions where the arts are integrated into regular nursing education, but not interventions limited to an examination context or self-contained art classes. Articles discussing the art of nursing, arts-based care methods, arts-based interventions in hospitals, or arts-based research methods in a nursing context are excluded.

#### 2.1.2. Outcomes

This review examines competence development, defined as the process of enhancing knowledge, skills, and attitudes required to effectively perform tasks [68], with a focus on generic competency domains in nursing, such as professional attitude, clinical care, communication, and collaboration [33]. Only research that pertains to these domains is included, while studies that solely focus on learning experience and learner satisfaction, as well as research on learning and examination stress, are excluded.

#### 2.1.3. Types of Studies

The review includes quantitative studies that enable the determination of causality between intervention and effect. It encompasses comparative studies with experimental or quasi-experimental designs, as well as non-experimental studies with a one-group pretest-posttest design [69]. Mixed-methods studies are included if they contain a relevant quantitative sub-study.

### 2.2. Literature Search and Screening

A systematic search for primary research studies was conducted in electronic databases relevant to nursing science, healthcare, and education. The databases searched were CINAHL, ERIC, Medline, PsycInfo, Scopus, and Web of Science. The Boolean phrase (nursing AND education OR nursing AND students) AND (art OR arts OR painting OR sculpture OR drawing OR music OR dance OR drama OR poetry OR photo* OR movie*) was applied to titles and abstracts. The full search strategy is displayed in Table S1. The database search was limited to articles with available abstracts and was supplemented by a manual backward search in relevant reviews [70].

To ensure the quality of the research, only peer-reviewed journal articles in English language published between January 1999 and December 2023, including electronic advance publications, were considered. This approach is in line with the growing body of relevant research since the mid-1990s [32]. Dissertations, book chapters, and other articles that might not have undergone independent review were excluded.

**Figure 1:**
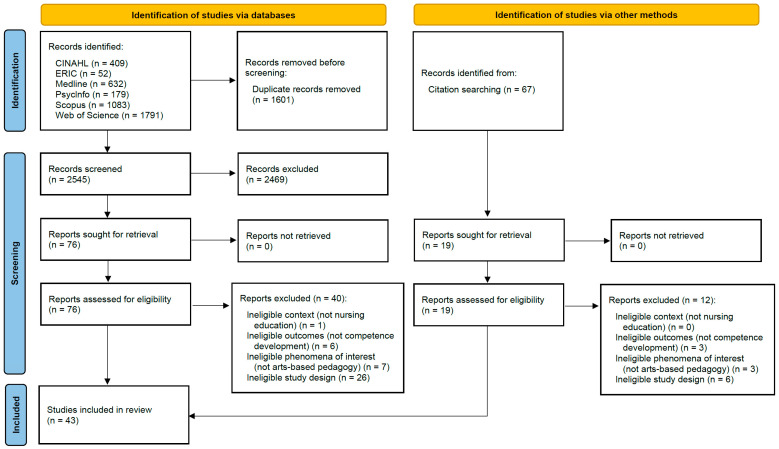
PRISMA flow diagram for literature search and selection.

The database and manual searches together yielded an initial 2612 potentially relevant articles. Subsequently, titles and abstracts were screened against the inclusion criteria, resulting in 95 articles in total for full-text screening. After the screening process, 43 articles remained for evaluation. Search outcomes are displayed in Figure 1, using standard PRISMA flow diagram [71]. Screening was conducted by the author and a second reviewer using a review software, the Joanna Briggs Institute System for the Unified Management, Assessment, and Review of Information (JBI SUMARI) [72]. The concordance for title and abstract screening was initially established at a rate of 99.5% (12 conflicts). In the event that a conflict could not be resolved through discussion, the reviewers included the relevant studies for further examination [70,73]. The full-text screening yielded a 100% match.

### 2.3. Quality Assessment

The Council for Exceptional Children (CEC) Standards for Evidence-Based Practices in Special Education [74] is the selected assessment tool for this review. Evidence assessment tools developed for health research are not entirely applicable to evaluate articles in education research [75]. The CEC Standards were chosen because they are specifically designed for pedagogical intervention studies and allow for a more rigorous appraisal than other approaches in education research [76,77,78].

The CEC Standards encompass important research on comparative studies and single-case research in the field, as presented by Gersten and colleagues [79], Horner and colleagues [80], and Lane and colleagues [81], as well as the criteria established by the What Works Clearinghouse (WWC) [82]. The CEC Standards are a common assessment tool in educational research. They are not exclusive to the field of special education [77], but they have also been used for systematic reviews in adult and higher education (e.g., [83,84,85,86,87]).

The CEC Standards guide the identification of evidence-based practices (EBPs) using 28 quality indicators (QIs) for the methodological soundness of group comparison studies and single-subject studies. The QIs cover eight areas: Context and Setting, Participants, Intervention Agents, Description of Practice, Implementation Fidelity, Internal Validity, Outcome Measures/Dependent Variables, and Data Analysis (Table 1). A study is considered sound if it meets all QIs in full. The CEC Standards also provide a grid for classifying the evidence base of practices based on high-quality research [82].

Quality criteria sets must be adapted to the context and scope of the systematic review [58]. For this review, QIs 2.2 and 5.3 of the CEC Standards were excluded because they refer to requirements in special education and do not fit arts-based pedagogy. QIs 6.6 and 8.2 were also excluded because they apply to single-subject studies only. QIs 6.4, 6.8, and 6.9 are only applicable to group comparison studies.

The CEC standards should only be applied to experimental studies that meet the criteria for EBP [74]. However, this review includes non-experimental studies to identify methodological challenges and promising approaches in arts-based pedagogy. To assess the methodological quality of non-experimental studies, the CEC checklist was slightly modified. QI 6.5, originally intended for single-subject studies, is considered to be met if the study used a pretest-posttest-follow-up design, because such a design provides information about the long-term impact, stability, and causal effects of the intervention, which enhances validity [88].

The author and a second reviewer independently assessed all studies for methodological quality using extensive guidelines for interpreting the QIs [79,80,81,82]. The scoring was recorded in a quality indicator matrix that followed the CEC Standards [89]. Inter-rater agreement was calculated within the matrix at the indicator level to demonstrate the reliability of quality appraisal. The interrater-agreement percentage was initially 98.9% on average for all articles. Any discrepancies were discussed and resolved by the reviewers through mutual agreement [70]. The assessment results are presented for both comparative and non-experimental studies in Appendix A in Table A1 and Table A2, respectively.

The CEC Standards require that all relevant QIs for the research design be met for a study to qualify as methodologically sound [74,82]. However, this benchmark has been criticized for being overly rigorous [81,90]. This review is based on a moderate understanding of evidence because, in educational research, it is appropriate to consider knowledge that does not correspond to the gold standard of evidence-based argumentation [91]. The scoring method suggested by Lane and colleagues [81] is followed, and QIs are weighed and given partial credit if met. A 80% cut-off point is applied to comparative studies. Studies that achieve 80% of QIs, equivalent to a score of 6.4, qualify as potential EBPs. Non-experimental studies, which represent a lower level of evidence than comparative studies [92], must meet the modified CEC Standards by 90%, equivalent to a score of 7.2.

### 2.4. Data Extraction

Data were extracted using summary tables for all comparative (Appendix A, Table A3) and non-experimental studies (Appendix A, Table A4). A concise summary is presented in Table 2. For mixed-methods studies, data extraction was limited to the characteristics of the quantitative sub-studies. The extraction was performed by the second reviewer and verified for accuracy in full by the author. The variables used for summarization were as follows: (a) intervention type, (b) study design, (c) participant characteristics and sample size, (d) data collection, (e) outcome measurements, and (f) key findings.

All studies were assessed for the certainty of evidence and categorized as having positive, mixed, neutral, or negative effects. The following criteria were established a priori [74,82]. Due to the heterogeneous nature of interventions and study designs, effect sizes were not taken into account. Studies are classified as having positive effects if statistically significant increases are demonstrated for all dependent variables. Studies are classified as yielding mixed effects if there are statistically significant increases in some dependent variables but not in others. Effects are classified as neutral if the intervention did not result in a statistically significant improvement in any of the dependent variables. If competencies deteriorate, the effect is termed negative.

### 2.5. Data Synthesis

To determine if arts-based pedagogy qualifies as an evidence-based practice (EBP), studies beyond the threshold of quality assessment are grouped based on comparable interventions in terms of art form, procedure, and outcome. A differentiated approach is required due to the heterogeneity of studies [133]. The study follows the evidence-based classifications established by CEC [74], and the results are presented in Table A5 in Appendix A.

According to CEC Standards, EBPs must demonstrate positive effects supported by a minimum of two robust group comparison studies with random assignment. As non-random assignment of participants to groups raises the risk of selection bias, the CEC Standards mandate that EBPs show positive effects backed by four methodologically sound group comparison studies [82]. A body of work that fails to meet the criteria for evidence-based practice may be categorized as “potentially EBP”, “mixed evidence”, “insufficient evidence”, or “negative effects” [74].

## 3. Results

After the screening process, 43 studies were included in the review. Out of these, 13 comparative studies met the criteria for a sound study and were evaluated as an EBP.

### 3.1. Participants and Settings

Most of the reviewed studies are based on data from undergraduate nursing students at universities or colleges. In six cases, participant groups were interdisciplinary, including medical or social work students [97,111,117,125,131,132]. Two studies took place in a professional training context [118,122]. Sample sizes for group comparison studies range from 40 to 267, while for non-experimental studies they range from 9 to 307.

### 3.2. Independent Variables

The studies reviewed cover a wide range of intervention designs that are based on various art forms.

A total of 15 interventions utilize the visual arts, with art observation being the most common design. Art observation is typically conducted in a museum (e.g., [56,102,115]). Three interventions engaged participants in creative assignments [95,103,113].

With a total of 10 studies, drama is a well-researched form of arts-based pedagogy [104,128,129]. Students typically participate in role-play or improvisation.

The sample includes five studies that used music as a teaching tool (e.g., [40,105]) or incorporated music into practical care [99]. Four studies examine the effects of cinenurducation [53] using movies as instructional material (e.g., [121,123]).

Other learning environments include photography [126], poetry [122], storytelling (e.g., [116]), comics and graphic novels [114], or a combination of different art forms [106,120].

### 3.3. Dependent Variables

Sensory perception skills are of particular interest for research (e.g., [56,102,106,107]) with a total of 10 studies. Above all, the impact of art observation on observation skills is examined. Five studies examine cognitive skills (e.g., [115,117,129]). Other studies focus on communication skills (e.g., [56,119]), diagnostic skills [102], or clinical skills [40,127].

Pedagogy that is based on the dramatic arts is often the subject of effectiveness research on attitudes. Ten studies examining the impact of arts-based pedagogy on attitudes toward others were analyzed (e.g., [96,104,116,131]), as well as five studies concerning attitudes toward other nursing issues (e.g., [112,128]). The research also covers complex concepts such as empathy, which is discussed in six studies (e.g., [93,122,132]), and professional identity, which is discussed in three (e.g., [103,121]).

Besides competence development, some studies examine personality traits such as self-efficacy [103], tolerance for ambiguity [110,111], and self-transcendence [95,113]. Five studies have explored the impact of arts-based pedagogy on knowledge acquisition, indicating that this is a peripheral research area (e.g., [114,116,129]).

### 3.4. Research Designs

Out of the 43 studies examined, 21 were group comparison studies, five of which were conducted as quasi-experiments. The remaining 22 studies were non-experimental. In the entire sample, eight studies utilized a mixed-methods design.

### 3.5. Methodological Quality

The quality appraisal results are presented in Appendix A in Table A1 for experimental and quasi-experimental studies and in Appendix A in Table A2 for non-experimental studies.

Of the reviewed studies, two experimental studies meet the Qis in full [104,128]. Eleven additional comparative studies score 80% or higher on the Qis [93,94,95,102,105,108,121,122,127,129,132]. Thirteen out of the twenty-one comparison studies achieved a high level of methodological quality, with a weighted score of 6.4 Qis or higher. The remaining eight comparison studies were of moderate quality, scoring at least 5.2 Qis.

Non-experimental studies did not meet the 90% threshold specified for this review, with six studies receiving a mediocre rating of 5.6 Qis or higher.

Common methodological shortcomings in all types of studies include inadequate definitions of dependent variables, a lack of reliability, and the absence of evidence of validity. Out of 22 comparison studies, nine lack reliability, and 14 lack evidence of validity (e.g., [114]). Out of the 21 non-experimental studies, 16 rely on measurement tools that use self-developed questionnaires, face validity, or scales that were transferred without reflection (e.g., [56,95,111,116,117,118]). While some measurement tools lack psychometric data, others require considerable effort to verify because they are referenced in articles that are not available in English [93,103,108,120,121,123,131].

Controlling for internal validity is a common issue in non-experimental studies. Twenty non-experimental studies used a pretest–posttest design, while two studies also conducted a follow-up test [98,125]. Several non-experimental studies have inaccuracies in data analysis and reporting (e.g., [100]). Other studies have problems with reporting implementation fidelity or exposure, or do not provide an in-depth description of the intervention (e.g., [119,125]).

### 3.6. Effects

Out of the 21 group comparison studies, 14 report significant positive effects on skill levels and attitudes. Three studies show mixed results, lacking significant effects on some dependent variables [95,102,103,113]. Four interventions had no impact [109,123,128]. Although arts-based pedagogy may have a positive impact on students’ competencies, it is not necessarily superior to conventional teaching. In two cases, researchers note positive effects but do not identify significant differences in competence development between the experimental and control groups [103,128].

Out of the 22 non-experimental studies, nine reported significant positive effects on nursing knowledge, skills, and attitudes. Another nine studies showed mixed results for changes in skill levels and attitudes. Four interventions failed to achieve an effect [96,97,117,119]. The outcomes are not related to art forms. Even comparable interventions may lead to different results [110,111].

## 4. Discussion

Out of the 43 studies reviewed, 13 are related to potential EBP as they achieve an 80% score for methodological soundness according to CEC Standards (Appendix A, Table A1). Three of these studies apply a quasi-experimental design [95,122,132], while ten meet the gold-standard of evidence by experimental design [69]. Ten studies describe arts-based interventions with positive effects on knowledge, skills, or attitudes (Appendix A, Table A5).

The sample is heterogeneous in terms of the studies included. It covers a range of different art-based interventions that must be assessed individually for each art form and targeted outcome when classifying research as evidence-based. Due to its variety, arts-based pedagogy needs to be evaluated less by “what works” but by “what works, for whom, and in what circumstances” ([133], p. 218).

### 4.1. Efficacy of Non-Dramatic Arts

A creative bonding intervention that employed students’ collages and other objects in practical care yielded mixed results on self-transcendence and attitudes toward elders [105]. A multi-week Visual Arts Training at a museum significantly improved participants’ observational skills but not their diagnostic competency [102]. The sample includes two musical interventions that positively impacted competence development. One intervention aimed to improve auditory skills [105], while the other utilized a disco song as an aid in cardiac resuscitation [127]. Two studies successfully introduced movies to the classroom [94,121]. Both studies screened movies without debriefing, but they differed in the number of movies shown and their duration. The experiments aimed to achieve different outcomes, with one focusing on empathy and the other on professional identity. One intervention that yielded positive effects, is based on poetry [122].

Pedagogical approaches to nursing education that are based on visual arts, music, movies, or poetry cannot be classified as evidence-based because an EBP requires at least one methodologically sound study to support it [82].

The use of visual arts training in museum settings to enhance perceptual abilities is a popular practice in nursing education and has garnered significant attention from researchers [31]. However, this approach has yet to yield robust research findings. Similarly, the incorporation of movies in teaching (cinenurducation) has inspired several studies [54], yet tangible research outcomes remain elusive. Arts-based learning offers interesting opportunities, such as exploring underrepresented art forms like comics, and developing interdisciplinary competencies such as intercultural skills [114]. Dance may enhance communication and collaboration skills [134] and other competencies relevant to clinical leadership [135], but it lacks solid quantitative research representation.

### 4.2. Efficacy of Creative Drama

Six studies aim to investigate the effects of drama-based pedagogy on nursing competencies [93,108,129,132], with two of them receiving the highest possible quality rating [104,128]. The learning experience was organized in a workshop format using creative drama. All workshops, except for one [129], were led by experienced or certified researchers in creative drama. Participants received training in drama techniques (e.g., [108]) or improvisation techniques [132] and actively applied them by reenacting [128] or role-playing typical care situations (e.g., [93]). One study demonstrates positive effects on postmortem care knowledge and skills [129]. Four studies report positive results on attitudes and empathy, while one intervention was found to be ineffective [128].

Three methodologically sound experimental studies on the use of drama in nursing education have reported positive effects on empathy and involved a total of 183 participants across studies [93,108,132]. These findings suggest that drama-based pedagogy qualifies as an evidence-based practice in nursing education according to the CEC classification [74]. However, it is important to note that these results are limited to empathy as a dependent variable, and there is insufficient evidence to support the effectiveness of creative drama in changing attitudes.

Creative drama is highly significant in nursing education and research because it allows students to explore complex nursing scenarios in a safe and supportive environment [47]. Empathy is an essential nursing competence because it fosters patient trust and the development of a successful therapeutic relationship [136,137]. Identifying creative drama as an EBP in terms of empathy adds to less rigorous research on the potential of drama in nursing education. Drama can enhance understanding of situations in clinical practice and the patient experience through fostering empathy and emotional engagement [48].

### 4.3. Impact on Professional Identity and Skills

Eight high-quality studies address attitudes reflecting the importance of professional values and their transmission in nursing education [138]. The arts-based teaching interventions documented in these eight studies successfully addressed altruism, empathy, and moral sensitivity (e.g., [93,94,121,122]). For healthcare professionals, prosocial behavior is crucial, and interpersonal competencies are essential in forming their professional identity [138]. As a potential trigger of deep reflection [27], arts-based pedagogy is an effective alternative to common approaches to identity formation, which is predominantly linked to traditional classroom learning [139].

Two high-quality studies focus on perceptual skills. They report positive effects of a music-based approach on auditory skills [105] and mixed effects of visual arts training on observational skills [102]. Together with inconclusive results from less rigorous studies (e.g., [56,107,109]), these findings challenge expectations for arts-based perceptual skills training in nursing education [31,46] and limit the meaningful scope of application to reflectivity. Visual arts have also been used in medical education to improve visual literacy and enhance students’ observational and diagnostic skills [140,141]. As in nursing education, there is a lack of robust evidence on the development of competencies [142,143,144,145,146].

### 4.4. Challenges and Implications for Research

The review supports previous findings that suggest a lack of methodological quality and rigor in research on arts-based nursing education [32,43]. Although there is a substantial body of literature, there is a clear lack of evidence to support the effectiveness of arts-based pedagogy in terms of competency development.

The results suggest a requirement for high-quality research on arts-based teaching methods. Nevertheless, there are various obstacles to implementing evidence-based practice in this area that subpar studies are unable to overcome convincingly. Arts-based practices pose a challenge to the standardization of interventions and replication [147]. Comparative studies may face difficulties in drawing generalizable conclusions due to variability in implementation fidelity, instructor expertise, and student engagement, which can introduce heterogeneity. Arts-based pedagogy is highly context-dependent and influenced by factors such as teachers’ attitudes and students’ experiences and preferences [27,148]. Contextual variables may interact with the intervention, making it challenging to isolate the effects of arts-based practices. Arts-based pedagogy encompasses a wide variety of artistic mediums, teaching approaches, and instructional strategies. Each practice may have unique characteristics, making direct comparisons between interventions difficult.

To assess the impact of arts-based interventions on competence development, reliable observational data and tested scales are necessary. Sound psychometry is needed to establish contemporary measurement tools for outcomes that arts-based methods typically address [149]. The high-quality studies examined in this review predominantly employ established measurement instruments. In medical education, there are a variety of quantitative scales for assessing observation skills, as well as psychometric scales used to assess the impact of arts-based pedagogies on ambiguity tolerance, communication skills, empathy, and mindfulness [150]. It is recommended that nursing education researchers prioritize the development, validation, and application of robust psychometric instruments tailored to arts-based educational interventions. This will ensure that future studies can more accurately measure and demonstrate the true impact of these pedagogies and their unique characteristics on nursing competencies.

### 4.5. Requirements and Implications for Educational Practice

Professional standards for nurse educator practice emphasize the importance of employing evidence-based approaches to curriculum design, choice of teaching strategies, and assessment methods [151]. The findings presented in this review suggest that educators expand their teaching repertoire, but integrate arts-based teaching methods with caution. While the potential benefits of arts-based pedagogy cannot be dismissed, the lack of robust evidence necessitates a measured approach. It is recommended that educators engage in ongoing professional development to refine their understanding and implementation of arts-based methods [152]. This should include training on how to effectively integrate these approaches into the curriculum and how to critically evaluate their impact on student learning and competence development. Furthermore, it is of paramount importance for nursing educators to advocate for and adhere to evidence-based practice [151]. This encompasses not only the application of research findings to practice but also the contribution to research itself [153].

### 4.6. Limitations

The quality appraisal is based on the CEC Standards and categorization scheme for EBP [74,82], with a less rigorous threshold applied [81]. The scope of the review and validity check is limited to English language publications. This approach to quality appraisal is not conclusive. Notably, the selection of quality evaluation tools impacts evaluation findings. Utilizing a different assessment tool and altering the weighting scheme will alter results [154]. Tools for assessing evidence specific to the social sciences are still deficient [75]. The field of education is currently engaged in intense debate about the definition of evidence and the standards that should be applied [91,147,155,156]. The concept of evidence in education extends beyond (quasi-)experimental findings. Unlike in medical science, which provides a variety of assessment tools, comparative studies are rare in educational research. Education is a social system with comparatively weaker validity [156]. As it falls into the category of the “harder-to-do sciences” ([147], p. 424), research on arts-based pedagogy requires specific standards for quality appraisal that do not yet exist.

This review examines the extent to which arts-based pedagogy improves the competencies of nursing students. It does not address the impact of arts-based pedagogy on the learning environment or other factors that contribute to learning success, such as learner engagement [157]. Successful arts-based pedagogy is largely based on disrupting behavior patterns and beliefs that facilitate the learning process [158,159,160]. Arts-based approaches are believed to benefit from experiential learning, multisensory learning, and emotional engagement [26,161,162]. However, their impact on learners and their influence on competence development require refined quantitative assessment methods and a wider range of methodologically sound comparative studies to build a more definitive evidence base for arts-based pedagogy.

## 5. Conclusions

Given the increasing recognition of non-traditional teaching methods in nursing education and the necessity to prepare students for the complexities of modern healthcare settings, research on arts-based pedagogy in nursing education is a growing area of interest. This research area is significant because it explores innovative teaching methods that can enhance nursing education and improve patient outcomes. However, there is a lack of evidence regarding the development of competence related to interventions and outcomes relevant to nursing practice, despite the variety of approaches stemming from different art forms.

This review aimed to evaluate whether arts-based approaches to nursing education improve nursing competence and meet the criteria for EBP. The review identified 43 quantitative studies that explored the impact of arts-based pedagogy on the knowledge, skills, and attitudes of nursing students. Thirteen comparative studies met the CEC Standards for high-quality research. Based on the CEC classification scheme, creative drama is considered an EBP, while other forms of arts-based pedagogy do not have enough sound studies to qualify as an EBP.

The findings suggest that the high expectations toward arts-based pedagogy in nursing education should be reconsidered in light of the evidence base. It is important to conduct high-quality research in this field to gain a better understanding of its effectiveness. This effort is critical to advancing arts-based pedagogy from an innovative educational experiment to a foundational, evidence-based practice in nursing education.

## Figures and Tables

**Table 1:** CEC quality indicators. Source: [74].

Quality Indicator		Description	
1.	Context and setting	1.1	Describes critical features of the context or setting (school or classroom)
2.	Participants	2.1	Describes participants’ demographics
		2.2	Describes disability or risk status and method for determining status
3.	Intervention agents	3.1	Describes role of the intervention agent, and background when relevant to review
		3.2	Describes agents’ training or qualifications
4.	Description of practice	4.1	Describes detailed intervention procedures and agents’ actions or cites accessible sources for that information
		4.2	Describes, when relevant, study materials described or cites accessible source
5.	Implementation fidelity	5.1	Assesses and reports implementation fidelity related to adherence with direct, reliable measures
		5.2	Assesses and reports implementation fidelity related to dosage or exposure with direct, reliable measures
		5.3	Assesses and reports implementation fidelity (adherence/dosage) throughout intervention and by unit of analysis
6.	Internal validity	6.1	Researcher controls and systematically manipulates independent variable
		6.2	Describes baseline or control conditions
		6.3	During baseline or control conditions, participants have no/extremely limited access to intervention
		6.4	Random assignment of groups
		6.8	Attrition is low across groups
		6.9	Attrition differential is low between groups or is controlled for
7.	Outcome measures/ dependent variables	7.1	Outcomes are socially important
		7.2	Defines and describes measurement of dependent variables
		7.3	Reports effects of intervention on all measures
		7.5	Provides evidence of adequate internal reliability
		7.6	Provides evidence of adequate validity
8.	Data analysis	8.1	Techniques are appropriate for detecting change in performance
		8.3	Reports appropriate effect size statistic(s) or provides data to calculate the effect size

**Table 2:** Summary of studies.

						No. of QIs Met	
Author	Intervention	Study Design	Sample	Outcome	PE	Abs.	Wt.
*Basit et al. (2023)* [93] Turkey	Drama	Exp.	n = 49	Altruism Empathy	•	6	7.67
*Briggs and Abell (2012)* [94] USA	Movies	Exp.	n = 49	Empathy	•	6	7.33
*Chen and Walsh (2009)* [95] Taiwan	Visual art	Quasi-exp.	n = 194	Self-transcendence Attitudes toward elders		5	7.00
Dickens et al. (2018) [96] UK	Movies	Non-exp. (MMD)	n = 66	Attitudes toward people with PBD Knowledge about people with PBD		4	6.33
Dingwall et al. (2017) [97] UK	Drama	Non-exp. (MMD)	n = 63	Attitudes toward older people		2	4.17
Eaton and Donaldson (2016) [98] USA	Drama	Non-exp.	n = 12	Attitudes toward older adults	•	5	7.00
Emory et al. (2021) [99] USA	Music	Non-exp. (MMD)	n = 18	Attitudes toward older adults		3	5.83
Gazarian et al. (2014) [100] USA	Digital storytelling	Non-exp.	n = 36	Patient advocacy		3	5.00
Grossman et al. (2014) [101] USA	Visual art	Non-exp.	n = 19	Mindfulness Observational skills	•	3	5.33
*Guo* et al. *(2021)* [102] China	Visual art	Exp.	n = 99	Observational skills Diagnostic skills		7	7.50
HadaviBavili and İlçioğlu (2024) [103] Turkey	Visual art	Exp.	n = 181	Attitudes and self-efficacy toward anatomy courses		4	5.50
*Hançer Tok and Cerit (2021)* [104] Turkey	Drama	Exp.	n = 40	Attitudes toward caring for dying patients	•	8	8.00
*Honan Pellico et al. (2012)* [105] USA	Music	Exp.	n = 78	Auditory skills	•	6	7.33
Honan Pellico et al. (2014) [106] USA	Visual art Music	Non-exp.	n = 23	Perceptual aptitude skill (auditory and visual)		3	5.67
Honan et al. (2016) [107] USA	Visual art Music	Non-exp.	n = 39	Perceptual aptitude skill (auditory and visual)		3	6.00
Ince and Çevik (2017) [40] Turkey	Music	Exp.	n = 73	Blood draw skills	•	4	6.33
*Kahriman et al. (2016)* [108] Turkey	Drama	Exp.	n = 48	Empathy	•	6	7.33
Kirklin et al. (2007) [109] UK	Drama	Quasi-exp.	n = 67	Observational skills		4	5.83
Klugman and Beckmann-Mendez (2015) [110] USA	Visual art	Non-exp.	n = 19	Tolerance of ambiguity Attitude toward communication Observational skills		2	4.00
Klugman et al. (2011) [111] USA	Visual art	Non-exp.	n = 32	Tolerance for ambiguity Observational skills	•	4	5.67
Kyle et al. (2023) [112] UK	Drama	Non-exp.	n = 175	Attitudes toward interprofessionalism and nursing advocacy	•	5	6.00
Lamet et al. (2011) [113] USA	Visual arts	Quasi-exp.	n = 98	Attitudes toward older people Self-transcendence Willingness to serve		5	6.00
Lesińska-Sawicka (2023) [114] Poland	Comics Graphic novels	Exp.	n = 62	Knowledge of cultural issues	•	4	6.17
Lovell et al. (2021) [115] USA	Visual art	Non-exp.	n = 218	Critical thinking (metacognitive awareness)	•	3	5.17
Moore and Miller (2020) [116] USA	Video storytelling	Non-exp.	n = 88	Knowledge, beliefs, and attitudes related to care for seriously ill people	•	3	5.00
Nash et al. (2020) [117] Australia	Drama	Non-exp. (MMD)	n = 65	Confidence and understanding in challenging situations		3	5.00
Nease and Haney (2018) [118] USA	Visual art	Exp.	n = 36	Observational skills Problem description and identification skills	•	3	5.17
Neilson and Reeves (2019) [119] UK	Drama	Non-exp. (MMD)	n = 100	Communication skills		3	3.67
Özcan et al. (2011) [120] Turkey	Misc.	Non-exp.	n = 48	Empathic skills	•	3	4.50
*Park and Cho (2021)* [121] South Korea	Movies	Exp.	n = 29	Professional nursing identity Professional nursing values	•	7	7.67
*Rashidi et al. (2022)* [122] Iran	Poetry	Quasi-exp.	n = 108	Moral sensitivity	•	6	6.83
Röhm et al. (2017) [123] Germany	Movies	Quasi-exp.	n = 51	Attitudes and social distancing toward stigmatized groups		3	5.83
Shieh (2005) [124] USA	Story writing Storytelling	Non-exp. (MMD)	n = 16	Nursing knowledge	•	4	5.50
Sinha et al. (2015) [125] USA	Visual art	Non-exp.	n = 36	Attitudes toward interprofessional collaboration Attitudes toward end-of-life care	•	1	1.83
Slota et al. (2018) [38] USA	Visual art	Non-exp.	n = 9	Observational skills Communication skills		4	5.17
Slota et al. (2022) [56] USA	Visual art	Non-exp.	n = 72	Observational skills Communication skills		3	4.67
Stupans et al. (2019) [126] Australia	Photo-essay	Non-exp. (MMD)	n = 77	Reflective thinking		3	4.83
*Tastan et al. (2017)* [127] Turkey	Music	Exp.	n = 77	Cardiac resuscitation skills	•	7	7.83
*Tokur Kesgin and Hançer Tok (2023)* [128] Turkey	Drama	Exp.	n = 78	Attitudes toward violence against women		8	8.00
*Uzun and Cerit (2023)* [129] Turkey	Drama	Exp.	n = 70	Postmortem care knowledge and skills	•	6	6.50
Wikström (2001) [130] Sweden	Visual art	Exp.	n = 267	Perception of good nursing care	•	4	5.67
Yamauchi et al. (2017) [131] Japan	Visual art	Non-exp.	n = 307	Attitudes toward people with mental health problems		5	6.67
*Zelenski et al. (2020)* [132] USA	Drama	Quasi-exp. (MMD)	n = 86	Interprofessional empathy	•	5	6.50

Note. Abbreviations: Misc. = miscellaneous; Exp. = experimental; MMD = mixed-methods design; PE = positive effect; Abs. = absolute; Wt. = weighted. Studies with authors highlighted in italics are considered high-quality based on CEC Standards [74].

## Supplementary Materials

The following supporting information can be downloaded at https://www.mdpi.com/article/10.3390/nursrep14020083/s1, Table S1: Database search.

## Public Involvement Statement

No public involvement in any aspect of this research.

## Guidelines and Standards Statement

Reporting follows the Preferred Reporting Items for Systematic reviews and Meta-Analyses (PRISMA) statement [71].

## Appendix A

**Table A1:** Quality assessment results for comparative studies.

	1.0	2.0		3.0		4.0		5.0			6.0									7.0						8.0			No. of QIs Met		Effects			
Author	1.1	2.1	2.2	3.1	3.2	4.1	4.2	5.1	5.2	5.3	6.1	6.2	6.3	6.4	6.5	6.6	6.7	6.8	6.9	7.1	7.2	7.3	7.4	7.5	7.6	8.1	8.2	8.3	Absolute	80% Weighted	Positive	Mixed	Neutral	Negative
Basit et al. (2023) [93]	•	•	NA	•	•	•	NA	NA	•	NA	•	•	•	•	NA	NA	NA		•	•	•	•	•		•	•	NA	•	6	7.67	•			
Briggs and Abell (2012) [94]	•	•	NA	•	•	•	NA	NA	•	NA	•	•	•	•	NA	NA	NA	•	•	•	•	•	•	•		•	NA		6	7.33	•			
Chen and Walsh (2009) [95]	•	•	NA	•	•	•	•		•	NA		•	•	•	NA	NA	NA	•	•	•	•	•	•			•	NA	•	5	7.00		•		
Guo et al. (2021) [102]	•	•	NA	•	•	•	•		•	NA	•	•	•	•	NA	NA	NA	•	•	•	•	•	•	•	•	•	NA	•	7	7.50		•		
HadaviBavili and İlçioğlu (2024) [103]	•	•	NA					NA	•	NA	•	•	•		NA	NA	NA	•	•	•		•	•	•		•	NA	•	4	5.50			•	
Hançer Tok and Cerit (2021) [104]	•	•	NA	•	•	•	NA	NA	•	NA	•	•	•	•	NA	NA	NA	•	•	•	•	•	•	•	•	•	NA	•	8	8.00	•			
Honan Pellico et al. (2012) [105]	•	•	NA	•	•	•		•	•	NA	•	•	•	•	NA	NA	NA	•	•	•	•	•	•	•		•	NA	•	6	7.33	•			
Ince and Çevik (2017) [40]	•	•	NA	•	•	•		•		NA	•	•	•	•	NA	NA	NA	•	•	•	•	•	•		•	•	NA		4	6.33	•			
Kahriman et al. (2016) [108]	•	•	NA	•	•	•	•	NA	•	NA	•	•	•	•	NA	NA	NA		•	•		•	•			•	NA	•	6	7.33	•			
Kirklin et al. (2007) [109]	•		NA	•	•	•	•	•	•	NA	•	•	•		NA	NA	NA	•	•	•		•	•			•	NA		4	5.83			•	
Lamet et al. (2011) [113]	•	•	NA	•	•	•	•			NA		•	•		NA	NA	NA			•	•	•	•			•	NA	•	5	6.00		•		
Lesińska-Sawicka (2023) [114]	•	•	NA		•	•	•	•		NA	•	•		•	NA	NA	NA		•	•		•	•			•	NA	•	4	6.17	•			
Nease and Haney (2018) [118]	•	•	NA		•			•		NA		•	•	•	NA	NA	NA			•	•		•	•		•	NA	•	3	5.17	•			
Park and Cho (2021) [121]	•	•	NA	•	•	•	•	•	•	NA	•	•	•	•	NA	NA	NA	•	•	•		•	•	•		•	NA	•	7	7.67	•			
Rashidi et al. (2022) [122]	•	•	NA	•	•	•	•			NA		•	•	•	NA	NA	NA	•	•	•	•	•	•	•	•	•	NA	•	6	6.83	•			
Röhm et al. (2017) [123]	•	•	NA		•		•	•		NA	•	•			NA	NA	NA		•	•	•	•	•	•		•	NA	•	3	5.83			•	
Tastan et al. (2017) [127]	•	•	NA	•	•	•	•	•	•	NA	•	•		•	NA	NA	NA	•	•	•	•	•	•	•	•	•	NA	•	7	7.83	•			
Tokur Kesgin and Hançer Tok (2023) [128]	•	•	NA	•	•	•	•	NA	•	NA	•	•	•	•	NA	NA	NA	•	•	•	•	•	•	•	•	•	NA	•	8	8.00			•	
Uzun and Cerit (2023) [129]	•	•	NA		•	•	•	NA		NA	•	•	•	•	NA	NA	NA	•	•	•	•	•	•	•	•	•	NA	•	6	6.50	•			
Wikström (2001) [130]	•		NA	•	•	•	•	•	•	NA	•	•	•	•	NA	NA	NA			•	•	•				•	NA		4	5.67	•			
Zelenski et al. (2020) [132]	•		NA	•	•	•	NA	•	•	NA	•	•	•		NA	NA	NA	•	•	•	•	•	•			•	NA	•	5	6.50	•			
Total	21/ 21	18/ 21	NA	16/ 21	20/ 21	18/ 21	13/ 21	10/ 21	14/ 21	NA	17/ 21	21/ 21	18/ 21	16/ 21	NA	NA	NA	14/ 21	18/ 21	21/ 21	16/ 21	20/ 21	20/ 21	12/ 21	8/ 21	21/ 21	NA	17/ 21						

Note. NA = not applicable.

**Table A2:** Quality assessment results for non-experimental studies.

	1.0	2.0		3.0		4.0		5.0			6.0									7.0						8.0			No. of QIs Met		Effects			
Author	1.1	2.1	2.2	3.1	3.2	4.1	4.2	5.1	5.2	5.3	6.1	6.2	6.3	6.4	6.5	6.6	6.7	6.8	6.9	7.1	7.2	7.3	7.4	7.5	7.6	8.1	8.2	8.3	Absolute	90% Weighted	Positive	Mixed	Neutral	Negative
Dickens et al. (2018) [96]	•	•	NA		•	•	•	NA	•	NA	•	•		NA				NA	NA	•	•	•	•	•		•		•	4	6.33			•	
Dingwall et al. (2017) [97]	•		NA		•	•	NA	NA		NA	•	•	•	NA				NA	NA	•		•	•			•		•	2	4.17			•	
Eaton and Donaldson (2016) [98]	•	•	NA	•		•	NA	NA	•	NA	•	•	•	NA	•	•		NA	NA	•	•	•	•			•	•	•	5	7.00	•			
Emory et al. (2021) [99]	•	•	NA		•	•	NA		•	NA	•	•	•	NA				NA	NA	•	•	•	•			•		•	3	5.83		•		
Gazarian et al. (2014) [100]	•	•	NA		•		NA	•		NA	•	•	•	NA				NA	NA	•	•	•	•	•	•	•			3	5.00		•		
Grossman et al. (2014) [101]	•	•	NA	•	•	•			•	NA		•	•	NA				NA	NA	•			•			•		•	3	5.33	•			
Honan Pellico et al. (2014) [106]	•	•	NA	•	•	•		•		NA	•	•	•	NA				NA	NA	•	•	•	•		•			•	3	5.67		•		
Honan et al. (2016) [107]	•	•	NA	•	•	•			•	NA	•	•	•	NA				NA	NA	•	•	•	•		•	•		•	3	6.00		•		
Klugman and Beckmann-Mendez (2015) [110]	•		NA	•	•	•		•		NA		•		NA				NA	NA	•		•	•			•			2	4.00		•		
Klugman et al. (2011) [111]	•		NA	•	•	•	•	•	•	NA	•	•		NA				NA	NA	•	•	•	•			•		•	4	5.67	•			
Kyle et al. (2023) [112]	•	•	NA	•	•	•	•			NA		•	•	NA				NA	NA	•	•	•	•	•	•	•		•	5	6.00	•			
Lovell et al. (2021) [115]	•	•	NA	•	•				•	NA	•	•		NA				NA	NA	•	•	•	•			•		•	3	5.17	•			
Moore and Miller (2020) [116]	•		NA	•	•	•	•	•		NA	•	•	•	NA				NA	NA	•				•		•		•	3	5.00	•			
Nash et al. (2020) [117]	•	•	NA		•	•	•		•	NA	•	•	•	NA				NA	NA	•		•	•						3	5.00			•	
Neilson and Reeves (2019) [119]	•		NA	•	•	•	NA			NA		•	•	NA				NA	NA	•			•						3	3.67			•	
Özcan et al. (2011) [120]	•	•	NA	•	•					NA		•	•	NA				NA	NA	•		•	•			•		•	3	4.50	•			
Shieh (2005) [124]	•		NA	•	•	•	NA	•	•	NA		•	•	NA				NA	NA	•		•	•			•		•	4	5.50	•			
Sinha et al. (2015) [125]	•		NA							NA		•		NA	•			NA	NA	•		•	•						1	1.83	•			
Slota et al. (2018) [38]	•		NA	•	•	•	•	•	•	NA	•	•	•	NA				NA	NA	•		•	•	•					4	5.17		•		
Slota et al. (2022) [56]	•		NA	•	•	•	•		•	NA	•	•	•	NA				NA	NA	•		•	•	•					3	4.67		•		
Stupans et al. (2019) [126]	•		NA	•	•	•	NA			NA		•	•	NA				NA	NA	•	•	•	•		•	•		•	3	4.83		•		
Yamauchi et al. (2017) [131]	•	•	NA	•	•	•	•	•	•	NA	•	•	•	NA				NA	NA	•		•	•			•		•	5	6.67		•		
Total	22/ 22	12/ 22	NA	16/ 22	20/ 22	18/ 22	8/ 22	8/ 22	12/ 22	NA	14/ 22	22/ 22	17/ 22	NA	2/ 22	1/ 22	0/ 22	NA	NA	22/ 22	10/ 22	19/ 22	21/ 22	6/ 22	5/ 22	16/ 22	1/ 22	15/ 22						

Note. NA = not applicable.

**Table A3:** Summary of comparative studies.

								Effects				No. of QIs Met	
Author	Intervention	Study Design	Participants	Data Collection	Outcome	Measurements	Key Findings	Positive	Mixed	Neutral	Negative	Absolute	80% Weighted
Basit et al. (2023) [93] Turkey	Drama Roleplay	Exp.	Nursing students n = 49	Q	Altruism Empathy	Altruism Scale Jefferson Scale of Empathy for Nursing Students (JSENS)	Significant increase in altruism and empathy No enduring effect	•				6	7.67
Briggs and Abell (2012) [94] USA	Movie	Exp.	Junior nursing students n = 49	Q	Empathy	Jefferson Scale of Physician Empathy (JSE)	Significant increase in empathy	•				6	7.33
Chen and Walsh (2009) [95] Taiwan	Visual art	Quasi-exp	Fourth-year nursing students n = 194	Q	Self-transcendence Attitudes toward elders	Self-transcendence scale (STS) Revised Kogan’s attitudes toward old people scale (RKAOP)	Significantly more positive attitude toward elders No effect on self-transcendence		•			5	7.00
Guo et al. (2021) [102] China	Visual art	Exp.	First-year nursing students in master program n = 99	Q	Observational skills Diagnostic skills	Clinical image test	Significant increase of observational skills Trend toward improvement of diagnostic skills		•			7	7.50
HadaviBavili and İlçioğlu (2024) [103] Turkey	Visual art	Exp.	First-year nursing and mid-wifery students n = 181	Q	Attitudes and self-efficacy toward anatomy courses	Anatomy attitude scale Anatomy self-efficacy scale	No significant effect			•		4	5.50
Hançer Tok and Cerit (2021) [104] Turkey	Drama Roleplay	Exp.	First-year Bachelor of Nursing Science students n = 40	Q	Attitudes toward caring for dying patients	Frommelt Attitude Scale for Caring for Dying (FATCOD)	Significantly more positive attitude toward dying patients	•				8	8.00
Honan Pellico et al. (2012) [105] USA	Music	Exp.	First-year nursing students in master program n = 78	Obs.	Auditory skills	N/A	Significant improvement of organ identification and sound interpretation	•				6	7.33
Ince and Çevik (2017) [40] Turkey	Music	Exp.	First-year nursing students n = 73	Obs.	Blood draw skills	Skill controls list	Significantly decreased anxiety levels Improved blood draw skills	•				4	6.33
Kahriman et al. (2016) [108] Turkey	Drama Roleplay Improvisation	Exp.	Practicing nurses n = 48	Q	Empathy	Empathic Skill Scale (ESS)	Significant increase in empathy	•				6	7.33
Kirklin et al. (2007) [109] UK	Drama	Quasi-exp.	Practicing nurses and doctors n = 68	Obs.	Observational skills	N/A	No significant effect			•		4	5.83
Lamet et al. (2011) [113] USA	Visual arts	Quasi-exp	Junior and senior nursing students n = 98	Q	Attitudes toward older people Self-transcendence Willingness to serve	Self-Transcendence Scale (STS) Attitudes toward Old People Scale	Significant improvement in attitudes toward older people Trend to increased willingness to serve		•			5	6.00
Lesińska-Sawicka (2023) [114] Poland	Comics Graphic novels	Exp.	First-year nursing students n = 62	Q	Knowledge of cultural issues	N/A	Significant increase in knowledge	•				4	6.17
Nease and Haney (2018) [118] USA	Visual art	Exp.	Practicing nurses n = 36	Obs.	Observation skills Problem description and identification skills	N/A	Significant improvement of observation skills Significant improvement of problem description and identification skills	•				3	5.17
Park and Cho (2021) [121] South Korea	Movies	Exp.	Second year undergraduate nursing students n = 29	Q	Professional nursing identity Professional nursing values	Perception of nursing checklist Professional nursing values scale	Significant improvement in perception of nursing and professional nursing values	•				7	7.67
Rashidi et al. (2022) [122] Iran	Poetry	Quasi-exp.	Practicing nurses n = 108	Q	Moral sensitivity	Nursing Moral Sensitivity Questionnaire (MSQ)	Significantly enhanced sensitivity	•				6	6.83
Röhm et al. (2017) [123] Germany	Movies	Quasi-exp.	Bachelor and master students in Rehabilitation Sciences n = 51	Q	Attitudes and social distancing toward stigmatized groups	Social Distance Scale Community Attitudes toward the Mentally Ill (CAMI)	No significant effect			•		3	5.83
Tastan et al. (2017) [127] Turkey	Music	Exp.	Second-year nursing school students n = 77	Obs.	Cardiac resuscitation skills	N/A	Significantly improved performance of cardiac resuscitation	•				7	7.83
Tokur Kesgin and Hançer Tok (2023) [128] Turkey	Drama Roleplay	Exp.	Fourth-year undergraduate nursing science students n = 78	Q	Attitudes toward violence against women	Violence Against Women Attitude Scale (ÍSKEBE)	No significant effect			•		8	8.00
Uzun and Cerit (2023) [129] Turkey	Drama Improvisation Roleplay	Exp.	Third-year undergraduate nursing science students n = 70	Q Obs.	Postmortem care knowledge and skills	Postmortem care knowledge test (PCKT) Postmortem care skills checklist (PCSCL)	Significantly improved postmortem knowledge and skill levels Enduring effect	•				6	6.50
Wikström (2001) [130] Sweden	Visual art	Exp.	First year nursing students n = 267	Q	Perception of good nursing care	Wheel Questionnaire	Significantly improved understanding of good nursing care	•				4	5.67
Zelenski et al. (2020) [132] USA	Drama	Quasi-exp. (MMD)	Students in health professions training programs (mainly nursing, pharmacy, medical) n = 86	Q	Interprofessional empathy	Interpersonal Reactivity Index (IRI) Consultative and Relational Empathy (CARE) Ekman Facial Action Coding System	Significant enhancement of interprofessional empathy	•				5	6.50

Note. Exp. = experimental; MMD = mixed-methods design; N/A = not applicable; Obs. = observation; Q = questionnaire.

**Table A4:** Summary of non-experimental studies.

								Effects				No. of QIs Met	
Author	Intervention	Study Design	Participants	Data Collection	Outcome	Measurements	Key Findings	Positive	Mixed	Neutral	Negative	Absolute	90% Weighted
Dickens et al. (2018) [96] UK	Movies	Non-exp. (MMD)	Undergraduate and postgraduate mental health nursing and counselling students n = 66	Q	Attitudes toward people with PBD Knowledge about people with PBD	Borderline Personality Disorder Questionnaire	Minor changes in knowledge and attitudes			•		4	6.33
Dingwall et al. (2017) [97] UK	Drama	Non-exp. (MMD)	Third-year nursing and social work students n = 63	Q	Attitudes toward older people	Self-developed questionnaire	Significant attitudinal changes among social work students only			•		2	4.17
Eaton and Donaldson (2016) [98] USA	Drama	Non-exp.	Second- and third-semester nursing students n = 12	Q	Attitudes toward older adults	Attitudes Toward Old People Scale (KOP) Refined Version of the Aging Semantic Differential (rASD)	Significant improvement in attitudes	•				5	7.00
Emory et al. (2021) [99] USA	Music	Non-exp. (MMD)	First-year bachelor nursing students n = 18	Q	Attitudes toward older adults	Perspectives on Caring for Older Patients (PCOP) Modified Kogan’s Attitudes toward Old People Scale (MKOP)	No significant effect for aggregate variables relating to attitudes toward older adults		•			3	5.83
Gazarian et al. (2014) [100] USA	Digital storytelling	Non-exp.	Senior-level nursing students n = 36	Q	Patient advocacy	Protective Nursing Advocacy Scale (PNAS)	Increase in perceptions of patient advocacy		•			3	5.00
Grossman et al. (2014) [101] USA	Visual art	Non-exp.	Nursing students n = 19	Q	Mindfulness Observational skills	Mindfulness Attention Awareness Scale (MAAS) Clinical Picture Assessment (CPA)	Significant improvement of mindfulness Significant improvement of observational skills	•				3	5.33
Honan Pellico et al. (2014) [106] USA	Visual art Music	Non-exp.	Fourth-year bachelor nursing students n = 23	Obs.	Perceptual aptitude skill (auditory and visual)	N/A	Improved observational skills Significant increase in auscultative interpretive skills		•			3	5.67
Honan et al. (2016) [107] USA	Visual art Music	Non-exp.	Students in an accelerated nursing master’s program for non-nursing college graduates n = 39	Obs.	Perceptual aptitude skill (auditory and visual)	N/A	Significantly improvement in most observational skills Significant increase in some auscultative interpretive skills No enduring effects		•			3	6.00
Klugman and Beckmann-Mendez (2015) [110] USA	Visual art	Non-exp.	Undergraduate and graduate nursing students, medical students n = 19	Q Obs.	Tolerance of ambiguity Attitude toward communication Observation skills	Variation of Budner’s Tolerance of Ambiguity Scale Communication Skills Attitude Scale (CSAS)	No significant effect on tolerance of ambiguity No significant effect on interest in communication Significant improvement of observational skills		•			2	4.00
Klugman et al. (2011) [111] USA	Visual art	Non-exp.	Undergraduate and graduate nursing students, different level medical students n = 32	Q Obs.	Observational skills Tolerance for ambiguity	Variation of Budner’s Tolerance of Ambiguity Scale Communication Skills Attitude Scale (CSAS)	Significant improvement in observational skills Significant increase in tolerance for ambiguity	•				4	5.67
Kyle et al. (2023) [112] UK	Drama	Non-exp.	Undergraduate nursing students n = 175	Q	Attitudes toward interprofessionalism and nursing advocacy	Attitudes toward Healthcare Teams Scale (ATHCTS) Protective Nursing Advocacy Scale (PNAS)	Significant improvement in attitudes toward interprofessionalism and nursing advocacy	•				5	6.00
Lovell et al. (2021) [115] USA	Visual art	Non-exp.	Traditional and accelerated first-year nursing students n = 218	Q	Critical thinking (metacognitive awareness)	Metacognitive Awareness Inventory (MAI)	Significant increase in metacognitive awareness	•				3	5.17
Moore and Miller (2020) [116] USA	Video storytelling	Non-exp.	Second-degree nursing students n = 88	Q	Knowledge, beliefs, and attitudes related to care for seriously ill people	Adapted Story Experience Questionnaire	Significant increase in knowledge, beliefs, and attitudes related to care for seriously ill people	•				3	5.00
Nash et al. (2020) [117] Australia	Drama Roleplay	Non-exp. (MMD)	Students from multiple health professions n = 65	Q	Confidence and understanding in challenging situations	Self-developed questionnaire	Increased confidence and understanding in challenging situations			•		3	5.00
Neilson and Reeves (2019) [119] UK	Drama	Non-exp. (MMD)	First-year nursing students n = 100	Q	Communication skills	Self-developed questionnaire	Improved communication skills			•		3	3.67
Özcan et al. (2011) [120] Turkey	Miscellaneous	Non-exp.	Third class and senior nursing students n = 48	Q	Empathic skills	Empathic Skill Scale	Significant increase in empathic skills	•				3	4.50
Shieh (2005) [124] USA	Story writing Storytelling	Non-exp. (MMD)	Associate Degree in Nursing students n = 16	Q	Nursing knowledge	Self-developed questionnaire	Significant improvement in five areas of nursing knowledge	•				4	5.50
Sinha et al. (2015) [125] USA	Visual art	Non-exp.	Mainly third-year nursing and medical students n = 36	Q	Attitudes toward interprofessional collaboration Attitudes toward end-of-life care	Self-developed questionnaire	Significantly improved attitude toward interprofessional collaboration Significantly improved attitude toward end-of-life care	•				1	1.83
Slota et al. (2018) [38] USA	Visual art	Non-exp.	Post-Master Doctor of Nursing Practice students n = 9	Q	Observation skills Communication skills	Self-developed Visual Intelligence Assessment Tool (VIA)	Significantly improved attitude toward the relevance of observational skills Improved observational skills Deteriorated communication skills		•			4	5.17
Slota et al. (2022) [56] USA	Visual art	Non-exp.	Post-Master Doctor of Nursing Practice and Clinical Nurse Leader graduate students n = 72	Q Obs.	Observational skills Communication skills	Self-developed Visual Intelligence Assessment Tool (VIA) Image Assessment	No change in overall visual intelligence scores Significant improvement of observational skills		•			3	4.67
Stupans et al. (2019) [126] Australia	Photo-essay	Non-exp. (MMD)	First year Bachelor of Nursing students n = 77	Q	Reflective thinking	Reflective Thinking Questionnaire	Significant increase in understanding and critical reflection Increase in reflection		•			3	4.83
Yamauchi et al. (2017) [131] Japan	Visual art	Non-exp.	Nursing students, social work students n = 307	Q	Attitudes toward people with mental health problems	Semantic Differential Attitude Scale regarding people with mental health problems	Significantly improved attitudes toward people with mental health problems		•			5	6.67

Note. Exp. = experimental; MMD = mixed-methods design; N/A = not applicable; Obs. = observation; Q = questionnaire.

**Table A5:** Summary of high-quality studies.

								Effects				No. of QIs Met	
Author	Intervention	Study Design	Participants	Data Collection	Outcome	Measurements	Key Findings	Positive	Mixed	Neutral	Negative	Absolute	80% Weighted
Chen and Walsh (2009) [95] Taiwan	Visual art	Quasi-exp.	Fourth-year nursing students n = 194	Q	Self-transcendence Attitudes toward elders	Self-transcendence scale (STS) Revised Kogan’s attitudes toward old people scale (RKAOP)	Significantly more positive attitude toward elders No effect on self-transcendence		•			5	7.00
Guo et al. (2021) [102] China	Visual art	Exp.	First-year nursing students in master program n = 99	Q	Observational skills Diagnostic skills	Clinical image test	Significant increase of observational skills Trend toward improvement of diagnostic skills		•			7	7.50
Briggs and Abell (2012) [94] USA	Movies	Exp.	Junior nursing students n = 49	Q	Empathy	Jefferson Scale of Physician Empathy (JSE)	Significant increase in empathy	•				6	7.33
Park and Cho (2021) [121] South Korea	Movies	Exp.	Second year undergraduate nursing students n = 29	Q	Professional nursing identity Professional nursing values	Perception of nursing checklist Professional nursing values scale	Significant improvement in perception of nursing and professional nursing values	•				7	7.67
Honan Pellico et al. (2012) [105] USA	Music	Exp.	First-year nursing students in master program n = 78	Obs.	Auditory skills	N/A	Significant improvement of organ identification and sound interpretation	•				6	7.33
Tastan et al. (2017) [127] Turkey	Music	Exp.	Second-year nursing school students n = 77	Obs.	Cardiac resuscitation skills	N/A	Significantly improved performance of cardiac resuscitation	•				7	7.83
Rashidi et al. (2022) [122] Iran	Poetry	Quasi-exp.	Practicing nurses n = 108	Q	Moral sensitivity	Nursing Moral Sensitivity Questionnaire (MSQ)	Significantly enhanced sensitivity	•				6	6.83
Basit et al. (2023) [93] Turkey	Drama Roleplay	Exp.	Nursing students n = 49	Q	Altruism Empathy	Altruism Scale Jefferson Scale of Empathy for Nursing Students (JSENS)	Significant increase in altruism and empathy No enduring effect	•				6	7.67
Hançer Tok and Cerit (2021) [104] Turkey	Drama Roleplay	Exp.	First-year Bachelor of Nursing Science students n = 40	Q	Attitudes toward caring for dying patients	Frommelt Attitude Scale for Caring for Dying (FATCOD)	Significantly more positive attitude toward dying patients	•				8	8.00
Kahriman et al. (2016) [108] Turkey	Drama Roleplay Improvisation	Exp.	Practicing nurses n = 48	Q	Empathy	Empathic Skill Scale (ESS)	Significant increase in empathy	•				6	7.33
Tokur Kesgin and Hançer Tok (2023) [128] Turkey	Drama Roleplay	Exp.	Fourth-year undergraduate nursing science students n = 78	Q	Attitudes toward violence against women	Violence Against Women Attitude Scale (ÍSKEBE)	No significant effect			•		8	8.00
Uzun and Cerit (2023) [129] Turkey	Drama Improvisation Roleplay	Exp.	Third-year undergraduate nursing science students n = 70	Q Obs.	Postmortem care knowledge and skills	Postmortem care knowledge test (PCKT) Postmortem care skills checklist (PCSCL)	Significantly improved postmortem knowledge and skill levels Enduring effect	•				6	6.50
Zelenski et al. (2020) [132] USA	Drama	Quasi-exp. (MMD)	Students in health professions training programs (mainly nursing, pharmacy, medical) n = 86	Q	Interprofessional empathy	Interpersonal Reactivity Index (IRI) Consultative and Relational Empathy (CARE) Ekman Facial Action Coding System	Significant enhancement of interprofessional empathy	•				5	6.50

Note. Exp. = experimental; MMD = mixed-methods design; N/A = not applicable; Obs. = observation; Q = questionnaire.
