# Supplementary material for: Effects of Arts-Based Pedagogy on Competence Development in Nursing: A Critical Systematic Review
Source: Nurs Rep. 2024 Apr 27;14(2):1089–118. doi: 10.3390/nursrep14020083 (PMC11130909; doi:10.3390/nursrep14020083)
Supplement: Supplementary file 1 [file nursrep-14-00083-s001.zip › nursrep-2919392-supplementary.pdf]

Table S1. Database search.

Last Run Via	Query	Limiters/Expanders	Results
Interface<br>- EBSCOhost Research Databases<br>Search Screen<br>- Advanced Search<br>Database<br>- CINAHL<br>Date<br>- 17.01.2024	TI (nursing education OR nursing students) ANDTI (art OR arts OR painting OR sculpture OR drawing OR music OR dance OR drama OR poetry OR photo* OR movie*) OR AB (nursing education OR nursing students) AND AB (art OR arts OR painting OR sculpture OR drawing OR music OR dance OR drama OR poetry OR photo* OR movie*)	Limiters<br>- Abstract Available; Publication Date:19990101-20231231; English Language; Peer Reviewed; Research Article<br>Expanders<br>- Apply equivalent subjects<br>Search modes<br>- Boolean/Phrase	409
Interface<br>- EBSCOhost Research Databases<br>Search Screen<br>- Advanced Search<br>Database<br>- ERIC<br>Date<br>- 17.01.2024	TI (nursing education OR nursing students) ANDTI (art OR arts OR painting OR sculpture OR drawing OR music OR dance OR drama OR poetry OR photo* OR movie*) OR AB (nursing education OR nursing students) AND AB (art OR arts OR painting OR sculpture OR drawing OR music OR dance OR drama OR poetry OR photo* OR movie*)	Limiters<br>- Academic (Peer-Reviewed) Journals; Published Date: 19990101-20231231; Journal or Document: Journal Article (EJ); Language: English<br>Expanders<br>- Apply equivalent subjects<br>Search modes<br>- Boolean/Phrase	52
Interface<br>- EBSCOhost Research Databases<br>Search Screen<br>- Advanced Search<br>Database<br>- MEDLINE<br>- 17.01.2024	TI (nursing education OR nursing students) ANDTI (art OR arts OR painting OR sculpture OR drawing OR music OR dance OR drama OR poetry OR photo* OR movie*) OR AB (nursing education OR nursing students) AND AB (art OR arts OR painting OR sculpture OR drawing OR music OR dance OR drama OR poetry OR photo* OR movie*)	Limiters<br>- Peer Reviewed; Publication Date:19990101-20231231; Abstract Available; English Language; Publication Type: Journal Article<br>Expanders<br>- Apply equivalent subjects<br>Search modes<br>- Boolean/Phrase	632
Interface<br>- EBSCOhost Research Databases<br>Search Screen<br>- Advanced Search<br>Database<br>- APA PsycInfo<br>Date<br>- 17.01.2024	TI (nursing education OR nursing students) ANDTI (art OR arts OR painting OR sculpture OR drawing OR music OR dance OR drama OR poetry OR photo* OR movie*) OR AB (nursing education OR nursing students) AND AB (art OR arts OR painting OR sculpture OR drawing OR music OR dance OR drama OR poetry OR photo* OR movie*)	Limiters<br>- Publication Year: 1999-2023; Publication Date: 19990101-20231231; Peer Reviewed; Publication Type: Peer Reviewed Journal; English language; Document Type: Journal Article<br>Expanders<br>- Apply equivalent subjects<br>Search modes<br>- Boolean/Phrase	179
Interface<br>- Hellenic Academic Libraries Link<br>Search Screen<br>- Advanced Search<br>Database<br>- Scopus<br>Date<br>- 18.01.2024	(TITLE-ABS-KEY (nurs* education) AND TITLE-ABS-KEY (art) OR TITLE-ABS-KEY (arts) OR TITLE-ABS-KEY (painting) OR TITLE-ABS-KEY (sculpture) OR TITLE-ABS-KEY (drawing) OR TITLE-ABS-KEY (music) OR TITLE-ABS-KEY (drama) OR TITLE-ABS-KEY (poetry) OR TITLE-ABS-KEY (dance) OR TITLE-ABSKEY (photo*) OR TITLE-ABS-KEY (movie))	PUBYEAR > 1998 AND PUBYEAR < 2024 AND (LIMIT-TO (SRCTYPE , "j")) AND (LIMIT-TO (DOCTYPE , "ar")) AND (LIMITTO (LANGUAGE , "English"))	1,083
Interface<br>- Web of Science<br>Search Screen<br>- Advanced Search<br>Database<br>- Web of Science Core Collection<br>Date<br>- 18.01.2024	nursing education OR nursing students (Title) AND art OR arts OR painting OR sculpture OR drawing OR music OR dance OR drama OR poetry OR photo* OR movie* (Title) OR nursing education OR nursing students (Abstract) AND art OR arts OR painting OR sculpture OR drawing OR music OR dance OR drama OR poetry OR photo* OR movie* (Abstract)	Article (Document Types) and English (Languages) Timespan: 1999-01-01 to 2023-12-31	1,791
